# Perspectives of Clinicians and Staff at Community-Based Opioid Use Disorder Treatment Settings on Linkages With Emergency Department–Initiated Buprenorphine Programs

**DOI:** 10.1001/jamanetworkopen.2023.12718

**Published:** 2023-05-10

**Authors:** Kimberly L. Sue, Marek Chawarski, Leslie Curry, Ryan McNeil, Edouard Coupet, Robert P. Schwartz, Christine Wilder, Judith I. Tsui, Kathryn F. Hawk, Gail D’Onofrio, Patrick G. O’Connor, David A. Fiellin, E. Jennifer Edelman

**Affiliations:** 1 Department of Internal Medicine, Yale School of Medicine, New Haven, Connecticut; 2 Yale Program in Addiction Medicine, Yale School of Medicine, New Haven, Connecticut; 3 Department of Emergency Medicine, Yale School of Medicine, New Haven, Connecticut; 4 Department of Psychiatry, Yale School of Medicine, New Haven, Connecticut; 5 Health Policy and Management, Yale School of Public Health, New Haven, Connecticut; 6 Friends Research Institute, Baltimore, Maryland; 7 Department of Psychiatry and Behavioral Neuroscience, University of Cincinnati College of Medicine, Cincinnati, Ohio; 8 Department of Medicine, University of Washington School of Medicine, Seattle; 9 Center for Interdisciplinary Research on AIDS, Yale School of Public Health, New Haven, Connecticut

## Abstract

**Importance:**

An increasing number of emergency departments (EDs) are initiating buprenorphine for opioid use disorder (OUD) and linking patients to ongoing community-based treatment, yet community-based clinician and staff perspectives regarding this practice have not been characterized.

**Objective:**

To explore perspectives and experiences regarding ED-initiated buprenorphine among community-based clinicians and staff in geographically distinct regions.

**Design, Setting, and Participants:**

This qualitative study reports findings from Project ED Health, a hybrid type 3 effectiveness-implementation study designed to evaluate the impact of implementation facilitation on ED-initiated buprenorphine with referral to ongoing medication treatment. Clinicians and staff from community-based treatment programs were identified by urban academic EDs as potential referral sites for ongoing OUD treatment in 4 cities across the US in a formative evaluation as having the capability to continue medication treatment. Focus groups were held from April 1, 2018, to January 11, 2019, to examine community OUD treatment clinician and staff perspectives on accepting patients who have received ED-initiated buprenorphine. Data were analyzed from August 2020 to August 2022.

**Main Outcomes and Measures:**

Data collection and analysis were grounded in the Promoting Action on Research Implementation in Health Services (PARIHS) implementation science framework, focusing on domains including evidence, context, and facilitation.

**Results:**

A total of 103 individuals (mean [SD] age, 45.3 [12.0] years; 76 female and 64 White) participated in 14 focus groups (groups ranged from 3-22 participants). Participants shared negative attitudes toward buprenorphine and variable attitudes toward ED-initiated buprenorphine. Prominent barriers included the community site treatment capacity and structure as well as payment and regulatory barriers. Perceived factors that could facilitate this model included additional substance use disorder training for ED staff, referrals and communication, greater inclusion of peer navigators, and addressing sociostructural marginalization that patients faced.

**Conclusions and Relevance:**

In this study of community-based clinicians and staff positioned to deliver OUD treatment, participants reported many barriers to successful linkages for patients who received ED-initiated buprenorphine. Strategies to improve these linkages included educating communities and programs, modeling low-barrier philosophies, and using additional staff trained in addiction as resources to improve transitions from EDs to community partners.

## Key Points

**Question:**

What are the perspectives and experiences regarding Emergency Department (ED) initiated buprenorphine among community-based clinicians and staff who provide follow-up opioid use disorder (OUD) treatment?

**Findings:**

In this qualitative study of 103 clinicians and staff at 4 community sites providing OUD treatment, participants voiced concerns with buprenorphine initiation in the absence of counseling services and in the ED; concerns about the ED as a treatment initiation site; difficulty accepting patients into buprenorphine treatment because of treatment capacity and payment issues; regulatory barriers; the need for more addiction-trained staff; the need for improved systems for communication between EDs and community partners; and means to address sociostructural needs of patients.

**Meaning:**

The findings reflect ways to improve care transitions for patients receiving initial buprenorphine treatment in EDs into community treatment sites, including staff training, improved communication, and focusing on patients’ sociostructural marginalization.

## Teaser

This qualitative study evaluates the perspectives of clinicians and staff at community-based opioid use disorder treatment settings on linkages with emergency department–initiated buprenorphine programs.

## Introduction

As opioid overdose deaths surge across the US,^1^ initiating buprenorphine treatment for patients with opioid use disorder (OUD) has never been more urgent. Establishing patients with OUD expediently on medication for opioid use disorder (MOUD), such as buprenorphine or methadone, is critical to reducing morbidity and mortality risk.^2^ Emergency departments (EDs) are critical settings for providing care for injection-related infections, overdose, and opioid withdrawal, as well as initiating buprenorphine for OUD.^3,4,5^

ED-initiated buprenorphine facilitates timely buprenorphine initiation^6^ and is cost-effective.^6,7^ However, patients receiving initial buprenorphine treatment in EDs face many potential barriers to ongoing MOUD in the community,^8^ and the linkage processes from EDs to community-based MOUD providers are not always effective. Several referral options exist, including linking patients to clinicians in primary care sites, opioid treatment programs (OTPs), substance use disorder treatment centers, or bridge clinics for low-threshold buprenorphine.^9,10,11,12^

However, 1 study^13^ analyzing pharmacy claims data demonstrated that only 28.5% of patients receiving initial buprenorphine treatment in an ED setting filled a subsequent prescription for buprenorphine from a community-based clinician. Thus, to inform implementation efforts to promote ED-initiated buprenorphine with receipt of ongoing treatment, we explored perspectives of community-based clinicians and staff positioned to potentially receive ED-based referrals for ongoing OUD treatment.

## Methods

### Overview of Project ED Health

Project ED Health was a multisite National Institute on Drug Abuse Clinical Trials Network–funded hybrid type 3 effectiveness-implementation study designed to evaluate the impact of implementation facilitation on ED-initiated buprenorphine with referral to treatment and involved focus groups as part of the formative evaluation.^14^ Project ED Health was approved by the Western institutional review board. This qualitative portion was part of the baseline preimplementation formative evaluation used to identify barriers and facilitators to tailor the larger facilitation strategy.^15,16^ Individuals who participated in focus groups provided verbal consent and received food during their focus group participation. We followed the Standards for Reporting Qualitative Research (SRQR) reporting guidelines.^17^ Data collection and analyses were grounded in the Promoting Action in Research Implementation in Health Services (PARIHS) framework.^18,19^

### Study Design and Setting

Project ED Health was conducted in 4 urban cities: Baltimore, Maryland; Cincinnati, Ohio; New York, New York; and Seattle, Washington. Clinicians and staff from 13 community-based treatment programs were identified by urban academic EDs as potential referral sites for ongoing OUD medication treatment among patients initiated on buprenorphine in EDs. Data collection occurred from April 1, 2018, to January 11, 2019. Sampling was designed to include sites with diverse treatment models and settings. One focus group was held at each community facility and included clinicians and staff based at federally qualified health centers, academic primary care centers, federally certified OTPs, and office-based opioid treatment programs. Also included were programs that, in addition to OUD treatment, provided managed withdrawal (eg, detoxification), and intensive outpatient program/dual diagnosis treatments. Data on race and ethnicity based on self report were collected and evaluated to better characterize group membership and representativeness of the participants.

### Data Collection and Measurements

Qualitative data collection occurred via focus groups with community-based addiction treatment clinicians and staff (groups ranged from 3-22 participants). Guided by PARIHS, focus groups examined the clinician and staff perspectives on evidence, context, and facilitation needs for accepting patients who had received ED-initiated buprenorphine (the focus group guide has been previously published).^14^ In-person focus groups were facilitated by study investigators who traveled to sites (K.H., G.D., P.G.O., D.A.F., E.J.E.), with 1 to 2 physician researchers (addiction medicine trained, not responsible for clinical care in any site location) per focus group, who used a standardized semistructured interview covering topics such as knowledge base of evidence, relationships with local EDs and referral processes for buprenorphine treatment. Focus groups were audio recorded and professionally transcribed. Participants were asked to complete a brief survey about demographics and training.

### Statistical Analysis

Focus group transcripts were imported into Nvivo version 12.7.0 (QSR International) and analyzed using a hybrid inductive and deductive approach to identify themes.^20,21^ The sample size was consistent with the number of focus groups typically needed to address a complex phenomenon and achieve thematic saturation.^22^ Research team members (E.J.E., K.H., R.M., K.S.) independently coded transcripts using inductive coding approaches according to principles of grounded theory to elicit initial patterns across the focus groups and different sites. A codebook was developed, agreed upon, and shared across all coders. Once all transcripts were coded, patterns and themes were discussed in group meetings and were nested in a deductive fashion into a priori topics. Finally, these codes were collated and mapped within the elements of PARIHS and iteratively discussed as a group and agreed upon as a team.^18,23^ Data were analyzed from August 2020 to August 2022.

## Results

### Themes

Characteristics of focus group participants are reported in Table 1. A total of 103 individuals (mean [SD] age, 45.3 [12.0] years; 76 participants were female and 64 participants were White) participated in 14 focus groups. Seven main themes emerged (Table 2). In the evidence domain, themes included: (1) concerns about buprenorphine as well as (2) variable attitudes toward the ED as a site that should initiate buprenorphine treatment. In the context domain, themes included: (3) community treatment site capacity and structure and (4) payment and regulatory barriers that disincentivized community buprenorphine provision. In the facilitation domain, themes included: (5) need for trained substance use disorder treatment staff, (6) need for improved referrals/communication across health care sites and systems, and (7) ability to address sociostructural marginalization.

**Table 1.** Participant Characteristics

Characteristic	Overall (N = 103)	Baltimore, MD (n = 40)	New York, NY (n = 12)	Cincinnati, OH (n = 22)	Seattle, WA (n = 29)
Age, mean (SD)^a^	45.3 (12.0)	45.5 (13.4)	45.0 (11.2)	45.5 (11.9)	44.9 (10.8)
Sex					
Men	27	12	4	4	7
Women	76	28	8	18	22
Race and ethnicity (participants able to select all that applied)					
American Indian or Alaska Native	1	0	0	1	0
Asian	6	1	1	2	2
Black	21	14	4	2	1
Hispanic	7	2	3	1	1
Native Hawaiian or Pacific Islander	0	0	0	0	0
White	64	24	4	12	24
Other^b^	3	0	2	0	1
NA (missing/invalid)	0	0	0	4	0
Current job title					
Clinician (MD, PA, NP)	22	2	3	5	14
Nurse and additional clinical staff (RN, MA)	15	3	4	2	9
Counselor (therapist, social worker, peer, recovery coach, health promotion advocate)	8	11	4	4	0
Administrator (nonclinician)	4	9	0	2	2
Other	7	1	1	4	2
NA (missing, invalid)	47	14	0	5	2

^a^ Available for 96 participants (5 missing, 2 invalid).

^b^ “Other” defined as all other racial and ethnic identities not included in White, Black, Asian, Native Hawaiian or Pacific Islander, American Indian or Alaska Native, or Hispanic.

**Table 2.** Illustrative Quotes Organized by PARIHS Element

PARIHS domain, theme	Illustrative quote (focus group)
Evidence	
Concerns about buprenorphine	“Yeah, but look at the small amount of people that might be ready...No, being around addicts for as long as I have, I would suppose that's what they would—many of them will do. It's a money-making thing. They can come and get it. Even while they highs [sic] can be on the street drugs, and they just come and get the Suboxone [buprenorphine-naloxone], going out and selling it.” (Site 3, OTP)
		Variable attitudes toward ED as appropriate site for treatment initiation	“We have a lot of people, like I said, wanna gas and go. They don't want a counsellor. They don't want no treatment. They don't want no groups. The emergency room doesn't have a cap on how many times those people can come. Then to me, they're gonna keep going to the ED to get Suboxone [buprenorphine-naloxone].” (Site 3, OTP)
“I think getting them started early as possible on medication-assisted treatment is important. Obviously, there's other components to it, such as counseling and psychosocial pieces, but I think if more patients could get taken care of in the hospital setting prior to coming to a treatment facility, it's only gonna better the care for the patient and make them more successful, most likely.” (Site 10, office-based addiction practice)		
Context	
Inflexible community treatment programs	“’Well, is that the ED? And they told me to come here.’ Like, that's it? It'll be like 11:30 in the morning, and we can't extend the walk-in hours, because the rules are the rules. Then we're like, ‘Well, sorry you have to come back tomorrow.’”
	“If they have active AH [auditory hallucinations], or they're psychotic, we typically turn them away, because we're not able to handle that here. If their medical issues are too severe, we also will turn them away. Once they've been referred and their referral is accepted, they go on my next open slot on my schedule. It may be tomorrow. It's only myself that does admissions, so it could be a week from now. It just depends on how many referrals have come my way.” (Site 3, OTP)
	“We are all pretty much at capacity, and none of us have space for new patients on our schedule, and…we also have to do the Suboxone, making sure they meet criteria, it can take an hour for us if this is a brand new patient.” (site 14/FQHC)
Payment and regulatory barriers as disincentives to buprenorphine provision	Additionally, on the individual patient level, some providers stated they would not agree to start a patient on medication if they did not have insurance: “Say they do, they come from the emergency room and we put some process in place, and they come down, if their insurance is not right, we can't put them on Suboxone [buprenorphine-naloxone]. If they have no insurance—they can't get on Suboxone [buprenorphine-naloxone]” (Site 3, OTP).
	“We're still under the umbrella of the hospital. We have to make the hospital happy. They are the ones that pay our bills. They are the ones that keep our lights on, and things like that. If they have all of these patients that are in in-patient…that they need to come out, and then we have these patients in the ED, who do you think they're gonna pick first? They're gonna pick the people that are in the MBU, the ITU [Intensive Treatment Unit] that needs to come out, because they are the ones that keep us afloat.” (Site 3, OTP)
	“People know who we are, it’s just been a very touchy thing about turf wars…I had, at one point, 26 docs, so we had the capacity for 3,000 people.” (Site 10, office-based addiction practice)
Facilitation	
Need for trained staff	“We desperately need a cadre of patient navigators just to keep people on the radar.” (Site 14, academic FQHC)
	Importance of peers: “A social worker is a great person, too, but I think a peer advocate, somebody who can talk to them right where they are…at their level of reading.” (Site 6, OTP).
Need for improved communication and referrals between ED and community	“I believe the numbers were 3 out of 80, they actually got in contact with and came for the initial treatment appointment.” (Site 9, academic addiction clinic)
	“We can give 'em a taper only [if they have no insurance]. Then that's another thing that happens in the ED, is a disconnect with what we accept and what they tell a patient is something…it’s a real disconnect.” (Site 3, OTP)
	“But that information wasn’t shared with him because it’s 2 different systems, which is a challenge…If that could become part of the project of checking to see if a patient was even remotely associated with an OBOT nurse so that we could be contacted or notified, that would be really helpful.” (Site 12, academic primary care practice)
Need to address sociostructural marginalization of patients	“Even in the ER, what they can do to provide a peer, and a peer can educate them along the way and guide them in the right direction. Because if the person don't know where the treatment center is at, how's they gonna go to the treatment center. One of the state requirements is that for anybody who's getting any type of medication assistance, after they leave the ER, they gotta have a…State ID, even to get suboxone or methadone (Site 3, OTP).”
	“I’ve been saying for a while now that it doesn’t work so hot when you just give 'em a bottle of Suboxone and a pat on the *ss and send 'em back out to homelessness and massive usage.” (Site 13, academic low barrier clinic)

Abbreviations: ED, emergency department; FQHC, federally qualified health centers; OBOT, office-based opioid treatment; OTP, opioid treatment programs; PARIHS, Promoting Action on Research Implementation in Health Services.

#### Concerns About Buprenorphine

Focus groups revealed ongoing concerns that some community-based clinicians and staff held about buprenorphine that presented barriers to smooth linkages in transition from EDs to community sites. Despite the Substance Abuse and Mental Health Services Administration recommendation that buprenorphine itself is effective for treating OUD, and should not be withheld or contingent upon counseling or receipt of other forms of psychosocial intervention,^24^ 1 person at site 3 (OTP) stated that “the Suboxone [buprenorphine-naloxone] is kinda like a Band-Aid on a bullet hole. They need the all-encompassing treatment to be able to be in recovery. The Suboxone [buprenorphine-naloxone] is just kinda a safety net for that.” In this case, the participant expressed concern that buprenorphine itself was not sufficient for OUD treatment, with counseling or other supports seen as a critical component of a treatment plan.

There was also fear that patients would divert or misuse ED-initiated buprenorphine. One person at site 3 (OTP) said: “They could say, ‘I’m going to the emergency room, get some Suboxone. Come on down and sell this. This is gonna help me to get the street drugs.’”

#### Variable Attitudes Toward ED-Initiated Buprenorphine

Attitudes toward the ED itself as a site of initiation for buprenorphine treatment for OUD were varied. Clinicians expressed concern that if patients potentially experienced precipitated withdrawal in this setting, it would be more difficult to engage them in future buprenorphine treatment attempts: “There was 1 case of maybe precipitated withdrawal…then, once they have that 1 bad experience, it’s really hard to get them onboard again” (site 13, academic low barrier clinic). On the other hand, some argued that starting treatment in the ED was a good thing: “If they are ready, it keeps 'em alive for 3 more days” (site 3, OTP).

Finally, there was concern generally that ED-initiated buprenorphine might attract patients who were not experiencing life-threatening emergencies. One participant at site 9 (academic addiction clinic) said, “It's giving the message that withdrawal is an emergency…It feels horrible, I'm sure it does, but it's not—you're not gonna die from opiate withdrawal. Is it appropriate to give it in the ER? I don't know.”

#### Community Site Treatment Capacity and Structure

Understanding the context of local health care delivery, health systems, and community buprenorphine treatment capacity was essential to the programs’ ability to receive patient referrals for buprenorphine from ED settings. Some programs had very specific hours of operation and initiation protocols for prospective patients and strict patient caps. One program required newly admitted patients to participate in counseling for 5 days without being provided MOUD so that the patient would prove motivation for treatment, even if a patient had received ED-initiated buprenorphine:

They would still have to be admitted to our program. The way our policies are set up, they have to be a complete patient of ours before we medicate them…They need an H and P [history and physical examination]. They need labs…If they sent them as a walk-in, which is what they have been doing, they don’t get same day admission. They still do the holding group, as if someone came in, walked off the street…The holding group is set up to show their motivation and their investment for treatment. Once they've been in holding group roughly about 5 days, then we admit them. We don't give them medication. (site 3, OTP)

Another bottleneck for smooth transitions to MOUD in the community was that 1 program started all patients with observed buprenorphine initiations, a protocol that can be staff- and time-intensive. One participant noted, “The only challenge is we only have 1 Saturday clinic a month, so we’re not gonna do a first-day [observed] induction on a Friday” (site 9, academic addiction clinic).

Other programs were open access or low-barrier models emphasizing schedule flexibility. One person at site 13, an academic affiliated aftercare clinic described “when we have a walk-in who’s probably in withdrawals right there, and on that moment, that cusp of making a decision to seek treatment, we do everything we can to see them, if we can, even the same day…The trick is to get them dialed into someone who’s waivered and has a moment to see them as soon as possible.” Several of these low-threshold programs noted that polysubstance use was not seen as a barrier to buprenorphine treatment and that additional polysubstance use was not a reason to discontinue buprenorphine, endorsing a harm reduction philosophy.

#### Payment and Regulatory Barriers

Payment methods and regulatory barriers were also important in transitions. Several participants remarked upon structures of payment models as a major barrier to their ability to provide care to more patients. One respondent noted they could not increase the patient census: “As it stands now, the [state] has a unique payment system. It's just hard to expand capacity…We've been sort of capped at volumes from a couple of years ago” (site 3, OTP).

Another participant noted that there were financial disincentives to provide the medication at the outpatient level: “If they’re in the emergency room and it’s Friday or Saturday, I think there’d be somebody who would say, ‘Well, we have beds in detox,’ as compared to, ‘Here’s a dose,’ which doesn’t pay very well compared to outpatient” (site 2, OTP).

#### Need for Trained Staff

Community-based OUD treatment clinicians had suggestions for facilitation opportunities including asking for more peer navigators or linkage social workers. There was also a request for specialized OUD trained staff in the ED: “We need more people that are trained in addictions in the ED, because that seems to not be the case” (site 3, OTP).

Many felt that more peers, social workers, or recovery coaches were needed to enhance capacity and communication across both the ED and the community sites. There was also a call for local champions navigating across health care systems on all levels. One participant praised a local buprenorphine physician champion: “He’s very active in engaging those community relationships, so I’m sure he had a piece in that getting out there in the community and linking those together from the hospital to the outpatient addiction center” (site 10, office-based addiction practice).

#### Improved Referrals and Communication

Another consistent theme across programs included the need for better bidirectional care coordination between the ED and community sites. Disparate electronic medical record systems were specifically noted as a barrier. Some ED clinicians printed out discharge summaries, while others called and left messages for programs.

However, successful linkages between the ED and community programs were possible. In one community treatment program, patients were encouraged to write thank you notes to fire departments and EDs to communicate successes to upstream partners that “the patient got into treatment” (site 10, office-based addiction practice).

Another participant noted the benefits of peer recovery coaches in facilitating smooth transitions:

The peer recovery coaches are instrumental in making this happen. I got here off the train at 7:00 in the morning and there's the peer recovery coach. I said, “What are you doing here?” He said, “I walked a patient over.” The patient was in the emergency department, waited till we were open…There's nothing more impressive than taking a patient from the emergency department and actually walking them across the street to treat.” (site 7, OTP)

#### Need to Address Sociostructural Marginalization

Many of the community-based treatment clinicians noted that social-structural marginalization experienced by patients, including poverty, homelessness and housing instability, and not being able to meet basic needs such as having an acceptable form of identification (eg, driver’s license) affected their perspectives on buprenorphine treatment. One clinician noted: “We’re basically looking, in the beginning, to stabilize our patients before we take that giant step in maintaining them because we’re not sure of their insurance, of their housing, the psychosocial aspect before we take on the role of maintaining our patients” (site 2, OTP). Homelessness and dual diagnosis mental health concerns were consistently mentioned. One clinician stated: “They don't know that services like ours exist. They think, “I can get meds, but how is that gonna help me with a place to sleep? Help me get my food stamps” (site 3, OTP). Focus group participants reiterated that while medication was important, solving these other structural forces associated with ongoing opioid use were equally important.

Transportation to and from appointments at the receiving community OUD treatment site was also a key barrier to transition to community programs. One person at site 8 (OTP) said: “Transportation-wise, if there’s money to say, ‘Hey, we’re gonna start you on buprenorphine. Let’s put you in a cab, put you in an Uber, get you to the place,’ so that you do your intake...” Individuals based at programs that had access to transportation vouchers felt this service was essential.

## Discussion

This qualitative evaluation of perspectives of community-based OUD treatment clinicians and staff regarding ED-initiated buprenorphine for individuals with OUD uncovered important barriers and facilitators from essential partners. Key themes that emerged within PARIHS domains included negative attitudes of focus group participants toward buprenorphine as an effective treatment for OUD without adjunctive counseling beyond that provided by the prescriber, concerns over the ED as a treatment initiation site, limited capacity to accommodate referred patients, payment and regulatory barriers, as well as the needs for more addiction-trained staff, improved communication between EDs and community partners, and means to address the sociostructural needs of patients.

Our findings build on previous studies^25,26^ that have examined how to strengthen the linkage from ED-initiated buprenorphine to community partners. On the ED side, studies^27,28^ have documented barriers and facilitators to ED-initiated buprenorphine; one study^29^ that interviewed both inpatient and outpatient clinicians implementing ED-initiated buprenorphine described referral incoordination, lack of a centralized referral agency, and outpatient clinicians noting lacking documentation of a visit and showing up without appointments. Another study surveyed community outpatient counselors about what could assist with buprenorphine treatment retention and they reported addressing social and structural factors as essential to engagement and retention.^30^

Importantly, many community treatment clinicians and staff viewed OUD treatment as a complex condition in which starting medication such as buprenorphine was fraught. Focus group participants had many concerns about prescribing the medication in the ED, inappropriate use of medication, lack of ability to access care that included counseling or other treatment modalities, and lack of ability to follow up at community sites if a patient’s social determinants of health were not addressed.

However, despite some barriers, our study highlights facilitators for promoting linkage of patients receiving ED-initiated buprenorphine to the community for ongoing MOUD. Specifically, facilitators of these linkages include low-threshold programs that will flexibly squeeze patients into an intake or evaluation to provide same day treatment on demand, having dedicated staff and financial resources to directly transport patients from the ED to community sites, champions across both sites facilitating communication, and programs that were able to address the sociostructural barriers to holistic improvement for OUD, including helping patients get identification cards, insurance, or housing. There was enthusiasm for dedicated peer navigators across 3 sites despite the lack of robust evidence.^31^

Future programmatic implementation should emphasize the education of existing addiction workforce staff on the evidence behind medication-first programs, in which provision of MOUD does not have restrictions or contingencies.^9,32,33,34^ When considering research, clinical and policy implications, it is important to know that successful and smooth linkages are possible^6,11,35^; however, health systems and programs must be flexible and accommodating, lowering barriers to entry to MOUD and addressing patients’ holistic needs. Linkage to care may be facilitated by recent regulatory changes.^36^ Telehealth continuation of buprenorphine is one promising possible intervention to provide follow-up MOUD without relying on traditional clinic models.^37^

### Limitations

This study has several limitations. The study took place in 4 urban centers, so it may not be generalizable to other urban, suburban, or rural locations. The study also might have selection bias and/or social desirability bias,^38^ as focus groups were scheduled concurrently with study investigator site visits. However, to mitigate this, interviewers were not from any of the study sites and participants were encouraged to share both positive and negative experiences.^39^ In addition, some members of the research team who were part of data design and analysis also led focus groups^40^; aware of the possible risk to objectivity, separate team members conducted the data analysis.

## Conclusions

Potential community-based addiction treatment partners to EDs offering buprenorphine initiation describe a lack of clear communication with EDs, concerns about buprenorphine, the appropriateness of the ED as a site for initiation, regulatory barriers, and being a part of programs that were inflexible. Focus group participants were additionally concerned about the provision of buprenorphine without simultaneously addressing the patient’s holistic social determinants of health needs as well as the need for streamlined linkage from social work or other health navigators. Our findings suggest that some community partners are open to ED-initiated buprenorphine linkages; however, there remain substantial educational and structural barriers. These linkages can be enhanced with streamlined communication, appropriate training and resources, and adoption of low threshold principles to provide continued MOUD treatment.

## Notes

Supplement.
Data Sharing Statement
