# Supplementary material for: Perspectives of Clinicians and Staff at Community-Based Opioid Use Disorder Treatment Settings on Linkages With Emergency Department–Initiated Buprenorphine Programs
Source: JAMA Netw Open. 2023 May 10;6(5):e2312718. doi: 10.1001/jamanetworkopen.2023.12718 (PMC10173026; doi:10.1001/jamanetworkopen.2023.12718)
Supplement: Supplement. — Data Sharing Statement [file jamanetwopen-e2312718-s001.pdf]

Data Sharing Statement

Sue. Perspectives of Clinicians and Staff at Community-Based Opioid Use Disorder Treatment Settings on Linkages with Emergency Department-Initiated Buprenorphine Programs. *JAMA Netw Open*. Published May 10, 2023. doi:10.1001/jamanetworkopen.2023.12718

Data
Data available: No
